# Complex Crater Collapse: A Comparison of the Block and Melosh Acoustic Fluidization Models of Transient Target Weakening

**DOI:** 10.1029/2024JE008544

**Published:** 2024-12-14

**Authors:** Hamish C. F. C. Hay, Gareth S. Collins, Thomas M. Davison, Andrea Rajšić, Brandon C. Johnson

**Affiliations:** ^1^ Department of Earth Sciences University of Oxford Oxford UK; ^2^ Department of Earth Science and Engineering Impacts and Astromaterials Research Centre Imperial College London London UK; ^3^ Department of Earth, Atmospheric, and Planetary Sciences Purdue University West Lafayette IN USA; ^4^ Department of Earth Environmental and Planetary Sciences Brown University Providence RI USA; ^5^ Department of Physics and Astronomy Purdue University West Lafayette IN USA

**Keywords:** impact cratering, acoustic fluidization, strengh weakening, hydrocode, complex crater

## Abstract

The collapse of large impact craters requires a temporary reduction in the resistance to shear deformation of the target rocks. One explanation for such weakening is acoustic fluidization, where impact‐generated pressure fluctuations temporarily and locally relieve overburden pressure facilitating slip. A model of acoustic fluidization widely used in numerical impact simulations is the Block model. Simulations employing the Block model have successfully reproduced large‐scale crater morphometry and structural deformation but fail to predict localized weakening in the rim area and require unrealistically long pressure fluctuation decay times. Here, we modify the iSALE shock physics code to implement an alternative model of acoustic fluidization, which we call the Melosh model, that accounts for regeneration and scattering of acoustic vibrations not considered by the Block model. The Melosh model of acoustic fluidization is shown to be an effective model of dynamic weakening, differing from the Block model in the style of crater collapse and peak ring formation that it promotes. While the Block model facilitates complex crater collapse by weakening rocks deep beneath the crater, the Melosh model results in shallower and more localized weakening. Inclusion of acoustic energy regeneration in the Melosh model reconciles required acoustic energy dissipation rates with those typically derived from crustal seismic wave propagation analysis.

## Key Points

- Acoustic fluidization is a mechanism to explain temporary weakening of target rocks during the collapse of large impact structures
- The Melosh model of acoustic fluidization that accounts for scattering and regeneration of acoustic energy is implemented in the iSALE code
- Regeneration of acoustic energy facilitates localized deformation and sustains vibrations for the duration of crater collapse

## 1 Introduction

Impact craters on many planetary surfaces are classified as simple or complex based on their size and internal morphology (Dence, 1965). Complex craters have flat floors, central massifs, terraced rim walls, and much lower depth‐to‐diameter ratios than their smaller, bowl‐shaped, simple crater counterparts (Pike, 1977). Remote‐sensing observations, geological mapping, geophysical evidence, and drill‐core data indicate that complex craters form by deep‐seated, gravity‐driven collapse of the rim walls and uplift of the crater floor (Dence et al., 1977; Kenkmann et al., 2012) that does not occur in simple craters. To explain the onset of complex crater collapse at the simple‐to‐complex transition, theoretical and numerical modeling of crater formation shows that a substantial reduction in the shear strength of the target rocks is required (Collins et al., 2002; McKinnon, 1978; Melosh, 1977; Wünnemann & Ivanov, 2003). However, the mechanism for this target weakening has long been debated (Kenkmann et al., 2012; Melosh & Ivanov, 1999). Proposed explanations for target weakening necessary to facilitate complex crater collapse include thermal softening (O’Keefe & Ahrens, 1993, 1999), shear heating (Crawford & Schultz, 2013; Dence et al., 1977), dynamic friction reduction (Senft & Stewart, 2009) and acoustic fluidization (Melosh, 1979; Melosh & Ivanov, 1999).

The most well‐developed hypothesis for target weakening during crater formation is acoustic fluidization. Inspired by strong ground motions recorded during explosion cratering (Cooper & Sauer, 1977; Gaffney & Melosh, 1982), this model supposes that seismic vibrations generated by the impact shock wave and scattered around the crater temporarily and locally reduce confining pressure—and hence frictional resistance—within the rock mass and permit shear deformation (Melosh, 1979). The time‐ and space‐averaged effect of the intermittent, random, localized deformation is that the sub‐crater rock mass behaves rheologically as a Bingham or viscous fluid until the vibrations subside (Melosh & Ivanov, 1999). The model elegantly explains the transient nature of weakening in predominantly cold rocks and how small‐scale, localized brittle deformation can produce large‐scale ductile deformation (Kenkmann et al., 2012; Riller et al., 2018).

Two mathematical descriptions of acoustic fluidization have been developed (Melosh & Ivanov, 1999): the original model (Melosh, 1979, 1996), which we refer to here as the “Melosh” model, and the Block (oscillation) model (Ivanov & Kostuchenko, 1997). While the Melosh model has been applied to the dynamic weakening of tectonic faults (Melosh, 1996) and the mobility of long‐runout landslides (Collins & Melosh, 2003), it has not previously been implemented in numerical simulations of crater formation. Instead, an alternative description of acoustic fluidization known as the Block model has been widely used in numerical simulations of large, complex crater collapse (e.g., Collins et al., 2002; Ivanov, 2005; Ivanov & Artemieva, 2002; Ivanov & Kostuchenko, 1997; Melosh & Ivanov, 1999; Wünnemann & Ivanov, 2003). Impact simulations that employ the Block model have been very successful at replicating the simple‐to‐complex transition and large‐scale morphometry of complex craters on a range of rocky and icy planetary bodies (e.g., Baker et al., 2016; Bray et al., 2014; Collins, 2014; Ivanov, 2019; Ivanov & Artemieva, 2002; Silber & Johnson, 2017; Wünnemann & Ivanov, 2003), the formation of peak‐ring craters on Earth (Collins et al., 2002; Collins, Morgan, et al., 2008; Ivanov, 2005) and the Moon (Kring et al., 2016), as well as the major structural deformation beneath large craters on Earth (e.g., Collins, Kenkmann, et al., 2008; Ivanov, 2005, 2020; Rae et al., 2017; Wünnemann et al., 2005). However, the region of weakening generated by the Block model is broad and unlocalized. As a result, discrete rim faults and terraced slump blocks are not produced by the Block model on its own; such features appear to require an additional strain localisation mechanism (e.g., Ivanov, 2021; Senft & Stewart, 2009), and so current simulations only accurately recreate the broad‐scale features of impact crater morphology. Moreover, it has proven difficult to relate the parameters of the block oscillation model to observations of fracture spacing and deformation at terrestrial drill cores (e.g., Rae et al., 2017), which may indicate that the Block model's prescription of the spatio‐temporal evolution of vibrational energy is too simplistic.

A key difference between the two models of acoustic fluidization is how they describe the evolution of acoustic energy during crater formation. In the widely adopted implementation of the Block model (Wünnemann & Ivanov, 2003), acoustic (vibrational) energy is generated by the expanding shock wave and then decays exponentially in time as the vibrations dissipate; diffusion and regeneration of acoustic energy, on the other hand, are neglected. In contrast, the Melosh model accounts for both scattering of acoustic energy and regeneration by deformation, as well as energy loss by dissipation (Melosh, 1996). Regeneration of acoustic energy may be particularly important, as this provides a feedback mechanism to promote strain localisation by prolonging transient pressure reduction in actively deforming zones or faults. Collins and Melosh (2003) showed that in the case of landslides self‐sustaining regeneration of acoustic energy during motion allowed large slides to travel long runout distances. However, compared to rock motion in large (>10‐km) crater collapse, large landslides are relatively thin (<1 km) and travel a large distance relative to their thickness. Thus, the confining pressure that must be compensated by vibrations is less and the distortional energy available for regeneration per unit volume is potentially greater for landslides than crater collapse. The importance of acoustic energy regeneration in crater collapse therefore remains unclear.

To explore the influence of acoustic energy regeneration and scattering on complex crater formation, here we describe the implementation of the Melosh model of acoustic fluidization into the iSALE shock physics code. We compare simulations of crater collapse that apply the Melosh model with those that apply the widely used Block model. We show that in the absence of scattering and regeneration of acoustic energy, the Melosh model of acoustic fluidization produces outcomes very similar to those of the Block model. However, when scattering and regeneration of acoustic energy are included, the kinematics of crater collapse are qualitatively different, with greater localisation, enhanced lateral collapse, and less deep‐seated uplift. In a companion paper, we show that the Melosh acoustic fluidization model can replicate the depth‐diameter progression of complex craters on Earth and the Moon and enable localized, fault‐like deformation near the crater rim (Rajšić et al., 2024).

## 2 Acoustic Fluidization Theory

Melosh (1979) introduced the theory of acoustic fluidization to explain the failure of geologic materials under surprisingly low differential stresses in the context of seismic faulting, long runout landslides and the collapse of large craters. The theory dictates that under the influence of a strong transient random acoustic wave field, a deforming fragmental rock mass exhibits the rheology of a Newtonian fluid with an effective viscosity $\eta_{\text{eff}}$ given by Collins & Melosh (2003); Melosh (1979); Melosh and Ivanov (1999):

(1) $$\eta_{\text{eff}}=\frac{\rho \lambda c_{p}}{2\psi }\left[\frac{2}{\text{erfc}\left(\chi \right)}-1\right],$$

where ρ is the density of the rock mass, λ is the wavelength of acoustic vibrations, and ψ is the ratio of the compressional to shear wave velocity squared, ${\left(c_{p}/c_{s}\right)}^{2}$. In this expression, the complementary error function, erfc, which here varies between 1 and 0, operates on the positive quantity $\chi =s_{c}/\sigma$, where $s_{c}=p-\tau /\mu$ is the critical vibrational pressure for grain sliding to occur given a driving stress τ, overburden pressure p and friction coefficient μ. The variance of the acoustic vibrational pressure, σ, is related to the acoustic vibrational energy density E by $\sigma =c_{p}\sqrt{2\rho E}$. When the energy in the acoustic wave field is large (small χ), the term in square parenthesis tends to unity and $\eta_{\text{eff}}=\frac{\rho \lambda c_{p}}{2\psi }$. When little acoustic energy is present (large χ), on the other hand, the effective viscosity becomes very large. Deformation of an acoustically fluidized rock mass is therefore a highly nonlinear function of the acoustic energy density of the wavefield and will depend critically on the spatial distribution of acoustic energy and its evolution in time.

The Block (oscillation) model (Ivanov & Kostuchenko, 1997; Melosh & Ivanov, 1999) is a widely used alternative to the Melosh model of acoustic fluidization (Melosh, 1979). Motivated by observations of large blocks disrupted by breccia in the deep drill core of the Puchez Katunki impact structure, it considers an analogous situation where a single block of length h oscillates with a period of T within a matrix of compressible breccia of density ρ. If the amplitude of oscillation is large enough, for a portion of the block's oscillation the normal stress of the block on the breccia beneath the block is reduced permitting the block to slip. This results in a simple rheological description of the rock mass that is similar to the rheology of an acoustically fluidized rock mass (Melosh & Ivanov, 1999). In this case, however, the vibrating rock mass is described by a Bingham rheology, with an effective yield strength given by:

(2) $$Y_{\text{eff}}=\mu \left(p-p_{\text{vib}}\right)+\eta_{\text{eff}}\dot{\epsilon },$$

where μ is the coefficient of friction, p is the overburden pressure, and $p_{\text{vib}}$ is the amplitude of the pressure vibrations from the oscillating blocks. The effective viscosity of the deforming rock mass is given by $\eta_{\text{eff}}=2\pi \rho h^{2}/T$ (Melosh & Ivanov, 1999). In this description, the block oscillation pressure can be related to an equivalent acoustic energy density E via $p_{\text{vib}}=c_{p}\sqrt{2\rho E}$, which controls the threshold yield strength in the Bingham rheology. Thus, the block oscillation pressure $p_{\text{vib}}$ of the Block model is equivalent to the variance of the acoustic vibrational pressure σ in the Melosh model.

According to both the Melosh acoustic fluidization model and the Block model, the rheology of a rock mass under the influence of strong vibrations is a nonlinear function of the vibrational or acoustic energy density E. Key to applying this rheological model to crater collapse is a description of the generation and evolution of acoustic energy following an impact. The initial source of acoustic energy is the impact‐generated stress wave. Recordings of the stress wave generated by explosions suggest that a long‐lasting coda of random stress wave energy persists behind the wave peak (Gaffney & Melosh, 1982). This vibrational or acoustic energy is caused by scattering and mode conversion as the wave interacts with small‐scale heterogeneities in elastic properties within the rock mass. To mimic this process without explicitly resolving subgrid‐scale variations in elastic properties, the implementation of the Block model in the iSALE shock physics code prescribes a cell‐centered vibrational particle velocity field $v_{\text{vib}}$ (proportional to the square root of the specific vibrational energy) as a fraction of the peak particle velocity achieved during expansion of the impact‐generated stress wave.

(3) $$v_{\text{vib}}=C_{\text{vib}}u_{p}$$

where $C_{\text{vib}}$ is a user‐defined constant, typically taken to be 0.1. To capture the peak particle velocity during generation of the shock wave, Equation 3 is applied for as long as it increases the vibrational velocity until a user‐prescribed time $T_{\text{off}}$ and up to a maximum vibrational velocity of 200 m/s. $T_{\text{off}}$ is conventionally set to the time taken for the shock wave to reach the simulation domain boundary so that reflected waves do not contaminate the vibrational velocity (energy) field.

According to the Melosh model of acoustic fluidization, the evolution of acoustic energy E is governed by the processes of dissipation, scattering and regeneration, which are described by the partial differential equation (Melosh, 1996):

(4) $$\frac{DE}{Dt}=\frac{\xi }{4}\nabla^{2}E-\frac{c_{p}}{\lambda Q}E+e\tau_{ij}{\dot{\epsilon }}_{ij},$$

where $\frac{D}{Dt}$ is the material derivative with respect to time t, ξ is the scattering diffusivity, e is the regeneration parameter, and Q is the dissipation quality factor, formally defined as the ratio of energy stored per cycle to the energy dissipated in that period (Collins & Melosh, 2003; Melosh, 1996). The first term on the right‐hand side diffuses (scatters) acoustic energy, with the strength of diffusion controlled by the scattering diffusivity, ξ. The second term describes the loss of acoustic energy by dissipation as heat. The acoustic wavefield dissipates faster as the product Qλ decreases. The last term accounts for the generation of acoustic vibrations (and energy) due to rock mass deformation. It enhances the acoustic energy field in areas undergoing large and rapid deformation. The total amount of distortional energy per unit time ($\tau_{ij}{\dot{\epsilon }}_{ij}$, the inner product of the stress tensor and the strain rate tensor) that can be converted to acoustic vibrations is controlled by the regeneration parameter e, which varies between zero and unity. The regeneration term introduces critical non‐linearity into the evolution of E because acoustic vibrations alter the effective viscosity of the rock mass (Equation 1) and, thus, its ability to deform. It is this term that provides the potential for acoustic fluidization to be self‐supporting. This term also provides a mechanism to generate the initial acoustic energy field in the wake of the impact‐generated stress wave in a manner similar to the way in which vibrational velocity is prescribed based on particle velocity in the Block model and with the same physical rationale.

In the implementation of the Block model in iSALE, the processes of scattering and regeneration of acoustic energy are neglected (ξ=0, e=0). In this case, the temporal evolution of acoustic energy is described by exponential decay $E=E_{0}\;\exp\left(-\frac{c_{p}t}{\lambda Q}\right)$, where $E_{0}$ is the initial acoustic energy density (after shock wave expansion). In terms of the vibrational particle velocity $\left(v_{\text{vib}}=\sqrt{2E/\rho }\right)$ used in the Block model in iSALE, which also decays exponentially, the decay time of vibrational velocity $T_{\text{dec}}$ is twice the decay time for acoustic energy: $T_{\text{dec}}=\frac{2\lambda Q}{c_{p}}$. This decay time is treated as a user‐defined constant in the Block model.

## 3 Implementing the Melosh Acoustic Fluidization Model in the iSALE Hydrocode

To implement the Melosh acoustic fluidization model in iSALE and incorporate the processes of scattering and regeneration of acoustic energy, we first modify Equation 4 so that it describes the evolution of acoustic energy per unit mass, $E=E/\rho =v_{\text{vib}}^{2}/2$. Moreover, as iSALE separates the full stress tensor into an isotropic part $p=-\text{tr}\left(\tau_{ij}\right)/3$ and a deviatoric part $\tau_{ij}^{\prime }=\tau_{ij}+p\delta_{ij}$, we also decompose the term describing the regeneration of acoustic energy into a volumetric and deviatoric contribution:

(5) $$\frac{\partial E}{\partial t}=\frac{\xi }{4}\nabla^{2}E-\frac{c_{p}}{\lambda Q}E+e_{s}\frac{\tau_{ij}^{\prime }{\dot{\epsilon }}_{ij}^{\prime }}{\rho }+e_{v}\frac{1}{\rho }\max\left(0,\frac{d\left(p+q\right)}{dt}\right),$$

where q is the artificial viscous pressure for numerical stability and ${\dot{\epsilon }}_{ij}^{\prime }$ is the deviatoric strain rate. Here we define two regeneration parameters for the shear $e_{s}$ and volumetric $e_{v}$ distortions and consider the generation of acoustic energy by compression but not expansion. This reformulation is optimal because iSALE stores specific energy rather than energy density and already advects the vibrational velocity field $v_{\text{vib}}$ for use in the Block model implementation. We also note that the (variance of) vibrational pressure for use in the rheological model (Equation 1) is expressed in terms of the specific acoustic energy as $p_{\text{vib}}=\sigma =\rho c_{p}\sqrt{2E}=\rho c_{p}v_{\text{vib}}$.

The scattering term in Equation 5 requires the Laplacian operator, which we add to iSALE for both cylindrical and cartesian coordinate systems. Except for the input parameters $e_{s}$, $e_{v}$, ξ, Q and λ, which may be specified at run‐time, the regeneration and dissipation terms depend on existing fields already stored by iSALE. These terms are thus straightforward to incorporate. Together, these modifications allow iSALE to calculate the terms on the right hand side of Equation 5, which are then used to step forward E in time. With this addition, we must also modify iSALE's internal energy evolution equation to ensure energy conservation.

Without acoustic fluidization, iSALE's conservation of internal energy equation in the Lagrangian step is:

(6) $$\frac{d\left(\rho I\right)}{dt}=\frac{d\left(p+q\right)}{dt}+\tau_{ij}^{\prime }{\dot{\epsilon }}_{ij}^{\prime },$$

where I is the specific internal energy. The terms on the right hand side represent the rate of volume work and distortional or plastic work, per unit mass, respectively. When acoustic fluidization is present, a small proportion of the volume and distortional work is converted into acoustic vibrational energy resulting in less internal energy. Conversely, as acoustic vibrations dissipate into heat, the internal energy increases. To account for this transfer between acoustic and internal energy, we modify Equation 6 so that the work terms account for internal energy lost to acoustic vibrations. An additional source term is also added to account for acoustic energy that returns to internal energy via dissipation. We thus alter iSALE's internal energy conservation to,

(7) $$\frac{d\left(\rho I\right)}{dt}=\frac{d\left(p+q\right)}{dt}-e_{v}\max\left(0,\frac{d\left(p+q\right)}{dt}\right)+\left(1-e_{s}\right)\tau_{ij}^{\prime }{\dot{\epsilon }}_{ij}^{\prime }+\frac{\rho c_{p}}{\lambda Q}E,$$

which reverts back to Equation 6 when acoustic fluidization is not present $\left(E=e_{v}=e_{s}=0\right)$. Together, Equations 5 and 7 control iSALE's energy conservation.

To account for the generation of the initial acoustic energy field by the expanding shock wave in an impact, we make use of the two acoustic energy regeneration terms. This avoids the need for an ad‐hoc prescription of vibrational particle velocity as a fraction of the peak particle velocity Equation 3, as adopted in the Block model implementation. Thus, as the shock wave propagates through the mesh a fraction of the dissipated pressure‐volume work and/or distortional energy is converted into acoustic energy, according to the $e_{s}$ and $e_{v}$ parameters.

Finally, to apply the rheological law of the Melosh acoustic fluidization model, we define an effective yield strength of the acoustically fluidized rock mass as:

(8) $$Y_{\text{eff}}=2\eta_{\text{eff}}{\dot{\epsilon }}_{\text{ps}}=\frac{\rho \lambda c_{p}}{2\psi }\left[\frac{2}{\text{erfc}\left(\chi \right)}-1\right]{\dot{\epsilon }}_{\text{ps}},$$

where ${\dot{\epsilon }}_{\text{ps}}$ is the second invariant of the deviatoric strain rate tensor and $\chi =\max\left(\left(p-\sqrt{J_{2}}/\mu \right)/p_{\text{vib}},0\right)$, is a measure of the pressure reduction necessary to induce slip under the current driving stress $\left(p-\sqrt{J_{2}}/\mu \right)$ relative to the amplitude of the pressure vibrations $p_{\text{vib}}$. To compute the former, the square root of the second invariant of the deviatoric stress tensor $J_{2}$ is used as the driving stress and, to be conservative, the coefficient of friction for the damaged material is used as the friction coefficient μ, which is assumed to be independent of pressure. We implement the acoustic fluidization rheology through an effective yield strength rather than as a viscosity for consistency with the Block model implementation and to avoid short momentum diffusion timescales caused by very high viscosities. This also ensures that the rock mass behaves elastically when the driving stress or vibrational pressure is low. The effective yield strength of the acoustically fluidized rock is capped at the static strength of the rock mass to ensure that acoustic fluidization does not artificially strengthen the rock mass.

For simulations involving multiple materials, separate acoustic fluidization parameters (λ, Q, ξ, $e_{s}$, $e_{v}$) are specified for each material but there is only a single, prognostic bulk specific acoustic energy field. In a mixed‐material cell, the contributions of each material to the specific acoustic energy update (Equation 5) are summed, weighted by the mass fraction of the material. Similarly, the effective yield strength of a mixed cell (Equation 8) is the volume‐weighted average of the effective yield strength of each material. All acoustic fluidization parameters are independent of depth.

## 4 Comparing the Melosh and Block Models of Acoustic Fluidization

### 4.1 Modeling Approach

To verify our implementation of the Melosh model of acoustic fluidization in iSALE and explore its similarity with the Block Model, we first consider a typical, simplified impact scenario on Earth that produces a complex crater. The impactor radius is 0.72 km, with a spatial resolution of 24 cells per projectile radius (CPPR), implying a cell dimension of 30 m, and the impact velocity is 12 km/s. To represent target and projectile material, we use a pressure‐, temperature‐ and strain‐dependent model of shear strength (Collins et al., 2004) and an ANEOS‐derived equation of state table (Pierazzo et al., 2008) for granite. Surface gravity is set to g = 9.81 m s^−1^. According to Collins et al. (2005), the expected morphology for these impact conditions is a complex crater with a rim‐to‐rim diameter of 17.8 km, and a depth of 0.7 km.

To serve as a reference, we first simulate this impact scenario using the Block model. In this simulation, the effective viscosity of material and decay time of acoustic vibrations are controlled by input parameters $\gamma_{\eta }$ and $\gamma_{\beta }$, respectively, for which we use previously recommended values for terrestrial craters (Table 1; Collins, 2014; Collins, Morgan, et al., 2008). Other input parameters controlling the evolution of acoustic vibrations in the Block model are the fraction of the peak particle velocity that initiates the vibrational velocity, $C_{\text{vib}}=0.1$, the time limit for shock‐induced vibrational velocity generation, $T_{\text{off}}=3$ s, and the maximum vibrational velocity, $v_{\text{vib,max}}=200$ m/s. All parameters are shown in Table 1.

**Table 1:** Block Model Input Parameters

Parameter	Description	Value
$\gamma_{\beta }$	Duration of vibrations	115
$\gamma_{\eta }$	Strength of vibrations	0.008
$\eta_{\text{eff}}$ ^a^	Effective viscosity (Pa s)	$7.5\times 10^{7}$
$T_{\text{dec}}$ ^a^	Decay time of vibrations (s)	16.56
$T_{\text{off}}$	Cutoff time	3 s
$C_{\text{vib}}$	Vibration velocity as a fraction of particle velocity	0.1
$v_{\text{vib,max}}$	Maximum vibrational velocity	200 m/s

^a^ In iSALE, $\eta_{\text{eff}}=\gamma_{\eta }a\rho c_{p}$ and $T_{\text{dec}}=\gamma_{\beta }a/c_{p}$, where a is impactor radius and $c_{p}=5000$ m/s.

### 4.2 Replicating Block Model Behavior

We then try replicating the Block model behavior using the Melosh acoustic fluidization model without scattering and regeneration. To match effective viscosity between the models, we take the complementary error function in Equation 1 to equal one because the material is strongly fluidized during cratering. The effective viscosity in the Block model is $\eta_{\text{eff}}=7.5\times 10^{7}$ Pa s. Thus, in the Melosh model, using ψ = 10 (Collins & Melosh, 2003; Rajšić et al., 2024), and ρ and $c_{p}$ for granite ANEOS, 2,629 kg/m^3^ and 5,476 m/s, respectively, the vibrational wavelength needed to match the Block model's $\eta_{\text{eff}}$ is λ=105 m. The user‐defined decay time of acoustic vibrations in the Block model is $T_{\text{dec}}=16.7$ s. To achieve an equivalent rate of acoustic energy dissipation in the Melosh model requires that $T_{\text{dec}}=\frac{2\lambda Q}{c_{p}}$, therefore the required dissipation factor is Q=431 (Table 2). Additionally, to ensure consistency between models, we include the same capping of maximum vibrational velocity ($v_{\text{vib,max}}=200$ m/s) and the same user‐prescribed time after which vibrations are no longer generated, $T_{\text{off}}=3$ s.

**Table 2:** Melosh Model Input Parameters

Parameter	Description	Value
λ	Wavelength of acoustic vibrations (m)	105.12
Q	Dissipation factor	431
$e_{v}$	Volume term	0.0025–0.01
$e_{s}$	Shear term	0–0.1
ξ	Scattering diffusivity ($m^{2}$/s)	0‐$3\times 10^{4}$
ψ	$c_{p}$‐to‐$c_{s}$ ratio	10

As the iSALE implementation of the Block model neglects scattering and regeneration of acoustic energy by shear deformation, we first demonstrate the behaviour of the Melosh acoustic fluidization model without these two terms (ξ=0, $e_{s}=0$). To generate acoustic energy from the expanding shock wave in a manner that is as consistent as possible with the Block model implementation, we assume a fraction $e_{v}$ of specific internal energy increase from PdV work, ΔI, is converted to specific acoustic energy, E: $e_{v}=E/\Delta I$. Conceptually, this mimics the process of acoustic energy generation in the wake of the shock wave attributed to the interaction of the wave with subgrid‐scale variations in elastic properties. To estimate the appropriate fraction, we calculate the specific internal energy increase during shock compression from the Hugoniot equation for energy conservation, $\Delta I=\left(P+P_{0}\right)\left(V_{0}-V\right)/2$. In the Block model, the vibrational velocity is calculated as a fraction of particle velocity, $C_{\text{vib}}=0.1$ (Equation 3), and capped at 200 m/s. We find the pressure and specific volume on the Hugoniot for granite at a particle velocity $u_{p}=2000$ m/s, which would result in the maximum vibrational velocity. The shock pressure where $u_{p}=2000$ m/s is P = $2.95\times 10^{10}$ Pa, and the change in specific volume from initial to shocked state, $V_{0}-V=1.35\times 10^{-4}$ $m^{3}$/kg, so $\Delta I=2\times 10^{6}$ J/kg. The specific acoustic energy associated with a vibrational velocity $v_{\text{vib}}=200$ m/s is $E=v_{\text{vib}}^{2}/2=2\times 10^{4}$ J/kg. Therefore, the required conversion of internal energy to acoustic energy, $e_{v}=0.01$.

**Figure 1:**
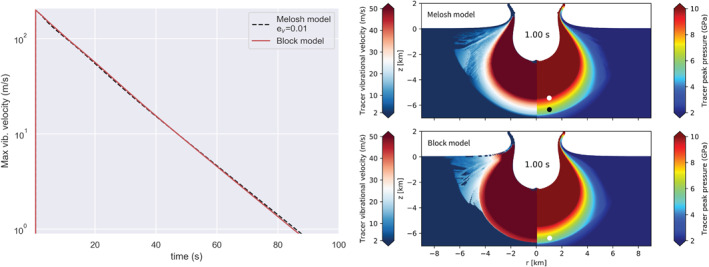
(left) Maximum vibrational velocity in the mesh as a function of time. The red solid line shows results from the block model, and the dashed black line shows results from the Melosh model simulation. (right) Vibrational field and peak pressure recorded in tracer particles 1 s after impact. The top simulation applied the Melosh model, and the bottom applied the Block model. The white dot marks the tracer particle where $V_{\text{vib}}<50$ m/s. In the Melosh model, this is where $P_{\text{sh}}$ = 9.5 GPa, while in the Block model, it is in the region where $P_{\text{sh}}$ = 6 GPa. For comparison, the black dot marks the region where $P_{\text{sh}}$ = 6 GPa in the Melosh model. Here, $V_{\text{vib}}=25$ m/s.

To verify the equivalence of the two models, we first compare the maximum vibrational velocity as a function of time in the Block and Melosh models. The values in Figure 1 (left) represent the maximum value measured across all cells in the high‐resolution zone (18×19.5 km). The red solid line represents the results from the simulation that applied the Block model, and the black dashed line represents the results of the simulations that included the Melosh model. When 1% of PdV work is converted to acoustic energy ($e_{v}$ = 0.01), the evolution of maximum vibrational velocity with time is nearly identical to the Block model.

**Figure 2:**
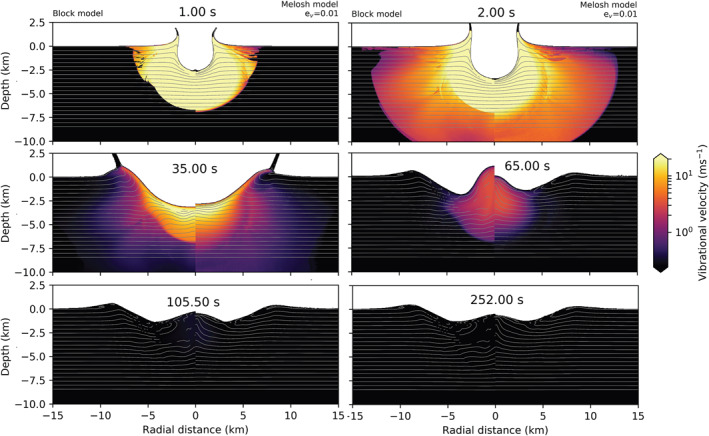
Time series showing the effect of the Block model (left from the origin) and the Melosh model (right from the origin) on crater collapse. The origin denotes the point of impact. The impact conditions are the same in both simulations (a=720 m, $V_{i}=12$ km/s). In the Melosh model, λ=105 m, Q=431, $e_{v}=0.01$, and $e_{s}=0$ and ξ=0.

In Figure 2, we show the evolution of the vibrational velocity field in simulations with the Block model (left from the origin) and the Melosh model ($e_{v}=0.01$; right from the origin). Qualitatively, the spatial distribution of the vibrational velocity field and its temporal evolution are similar in both simulations, leading to the development of very similar final craters. A fluidized zone with radially decreasing vibrational velocity expands over the first few seconds and then contracts as the magnitude of the vibrational velocity declines. At all times, the approximate size of the fluidized region and the maximum vibrational velocity are approximately the same in both simulations. However, radial profiles of vibrational velocity show a noticeable difference between the two simulations. In the Melosh model simulation, vibrational velocity declines smoothly with radius, whereas in the Block model simulation, there is an abrupt jump or discontinuity in the vibrational velocity field at a radius of ∼5.4 km that separates an inner, more intensely fluidized region from an outer, less fluidized zone until a time of about 35 s.

Although the maximum vibrational velocity has the same evolution with time between the two models (Figure 1, left), the spatial distribution of vibrational velocity is different even at early times (Figures 1 and 2, right). We attribute this difference to the different ways in which the vibrational velocity is generated in the two approaches: as a direct conversion from particle velocity in the Block model and via pdV work conversion in the Melosh model. The region of more intense vibrations ($v_{\text{vib}}\geq 50$ m/s) within a radius of ∼5.4 km corresponds to the area where the shock pressure $P_{\text{sh}}$ greatly exceeds the yield strength Y. Within this region, distortional energy does not contribute to the energy balance, implying that the particle velocity depends only on the isotropic PdV work. Beyond this distance from the impact, however, $P_{\text{sh}}$ rapidly drops to a few GPa (Figure 1, right), and the distortional energy contribution cannot be neglected $\left(P_{\text{sh}}∼Y\right)$. In other words, at these lower pressures, the particle velocity is augmented by strength effects and hence is larger than that derived solely from the isotropic PdV work.

A consequence of the subtle difference in the spatial distribution of vibrational energy is that the transient crater in the Block model simulation is somewhat deeper and narrower than the transient crater in the Melosh model simulation (t=35s), and subsequent floor uplift result in a taller, more prominent overheightened, central uplift (t=65s). Nevertheless, the final crater morphology in the two simulations is very similar. The final crater in the Block model simulation has a rim‐to‐rim crater diameter $D_{f}$ = 17.94 km (*t* = 252 s), whereas the crater produced in the Melosh model simulation was 3.6% smaller (640 m) in diameter. Both models have similar depth‐to‐diameter ratios (d/D): the Block model d/D=0.1, and the Melosh model d/D=0.09. The height of the central uplift is comparable between the models, while the rim‐to‐floor depth differs by 13.6% (240 m). The main difference in the final crater morphometry is, therefore, the crater rim height and floor depth.

### 4.3 Effects of Including Scattering Term

Next, we explore the effect of the scattering term in the Melosh model. A scattering timescale is defined as $T_{\mathrm{scatter}}=a^{2}/\xi$, where a is the impactor radius (a=0.72 km). To have $T_{\mathrm{scatter}}$ comparable to $T_{\text{dec}}=16.7$ s, we set the scattering diffusivity to $\xi =3\times 10^{4}m^{2}$/s, which is an order of magnitude larger than previously explored in Collins and Melosh (2003). A wider range of scattering diffusivities are explored in our companion paper (10–$10^{5}$ m^2^/s) (Rajšić et al., 2024).

**Figure 3:**
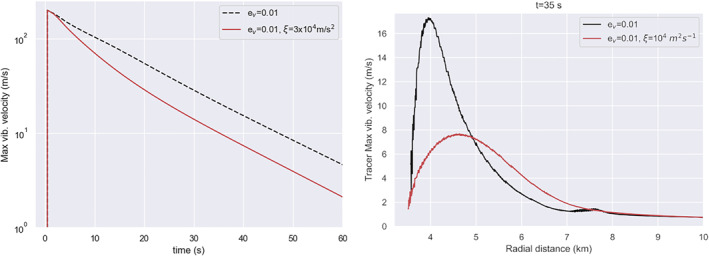
(left) Maximum vibrational velocity in the mesh as a function of time. Shown in the red solid line are results from a simulation with $\xi =3\times 10^{4}$ m^2^/s, and in the black dashed line are results from a simulation with ξ=0. (right) Vibrational velocity as recorded in tracer particles at 45deg from the impact point. In red is shown simulation with ξ=0, and in black simulation with $\xi =3\times 10^{4}$ m^2^/s.

First, we compare maximum vibrational velocity as a function of time between simulations with and without scattering (Figure 3, left). All other parameters were the same between simulations (λ=105 m, Q=431, $e_{v}=0.01$). In Figure 3, the dashed black line corresponds to the simulation with ξ=0 (c.f., Figure 2). Including scattering results in a decrease in the maximum vibrational velocity as a function of time. Additionally, we compare the depth distribution of vibrational velocity at several time steps (Figure 3, right). The same vibrational velocity field is generated in the first second after impact. As the simulation progresses, the spatial distribution of vibrational velocity evolves differently between the two cases as acoustic energy diffuses away from the impact site in the case where scattering is included. After 35 s, the vibrational velocity is half as large at the same depth (3–4 km from impact point) in the case that includes scattering ($v_{\text{vib}}=10$ m/s). At later time steps (t=65 and 105 s), lower vibrational velocity is still present in the simulation that includes scattering.

**Figure 4:**
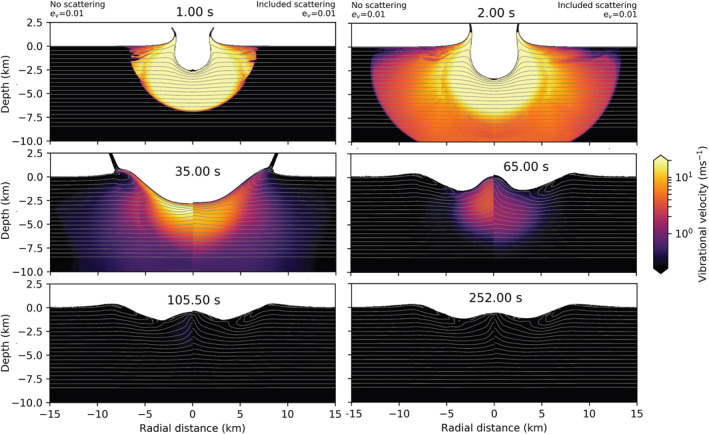
Time series showing the effect of the scattering term on crater collapse and evolution of the vibrational field. Left from the origin, $\xi =3\times 10^{4}m^{2}$/s, and right, ξ = 0. The origin denotes the point of impact. The impact conditions are the same in both simulations (a = 720 m, $V_{i}$ = 12 km/s). In both models λ = 105 m, *Q* = 431, $e_{v}$ = 0.01, and $e_{s}$ = 0.

The difference in spatio‐temporal evolution of vibrational velocity is also shown in Figure 4. Initially, during shock propagation and initial crater growth, neglecting or including scattering does not dramatically affect the spatial distribution of vibrations (top two frames). However, at later times (t=35–65 s), during floor uplift, vibrations diffuse away from the fluidized region in the simulation that includes scattering, resulting in a lower magnitude of the vibrational velocity within the important crater collapse zone (also, Figure 3, right). However, these changes do not substantially affect the final morphology of the crater (last frame; *D* = 17.3 km; *d* = 1.5 km).

### 4.4 Effects of Including Shear (re)generation

Next, we explore the effect of introducing shear (re)generation of acoustic energy. As introducing this additional term increases the total acoustic energy available for fluidization, it has a more profound effect on the evolution of acoustic energy and the kinematics of crater collapse. We therefore consider a suite of scenarios where we progressively increase the shear regeneration coefficient $e_{s}$ up to the previously recommended value for shear regeneration ($e_{s}$ = 0.1; (Collins & Melosh, 2003)) while reducing the volume regeneration coefficient $e_{v}$. In this set of simulations we set $T_{\text{off}}$ to the end of the simulation time. Considering that shear regeneration is supposed to generate vibrations for as long as there is deformation in the target (Equation 4), including $e_{s}$ while limiting the time for generating vibrations $\left(T_{\text{off}}\right)$ contradicts the purpose of the term and its effects on crater collapse.

**Figure 5:**
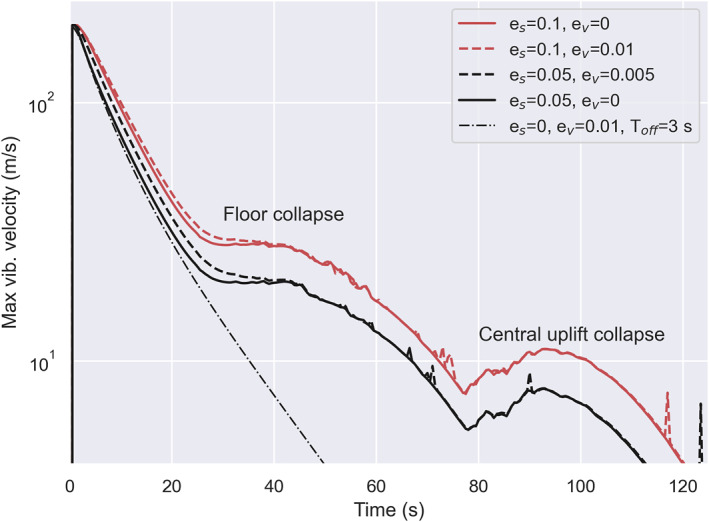
Maximum vibrational velocity in the mesh as a function of time. Solid lines correspond to cases where $e_{v}$ = 0, and dashed lines where $e_{v}>$0. Red lines correspond to cases where $e_{s}$ = 0.1, and black lines to cases where $e_{s}$ = 0.05. Dash‐dotted line correspond to the simulation without $e_{s}$. All simulations included ξ = $3\times 10^{4}$ m^2^/s.

In Figure 5, we compare the maximum vibrational velocity as a function of time between several scenarios. All scenarios included scattering with $\xi =3\times 10^{4}$ m^2^/s. In red, we show simulations that included only $e_{s}$. In black are shown scenarios which varied both $e_{s}$ and $e_{v}$. The dash‐dotted black line shows a simulation from the previous section, where $e_{s}=0$ and $e_{v}=0.01$. When shear regeneration is neglected, generated vibrations exponentially decay as a function of time. Including shear regeneration changes the behavior of the maximum vibrational velocity. Up until ∼30 s strong vibrations are decaying exponentially as a function of time. However, with the onset of the floor collapse, the maximum vibrational velocity significantly differs. The vibrations get regenerated between ∼30–80 s, and again between ∼ 80–120 s during the collapse of the central uplift.

When comparing the simulations that considered only $e_{s}$ (solid lines) with simulations that included both $e_{s}$ and $e_{v}$ (dashed lines), we see that the difference in maximum vibrational velocity evolution with time is minimal. Minor differences in the amplitude of maximum vibrational velocity occur during crater growth (∼ 0–40 s). However, maximum vibrational velocity regeneration is the same during crater collapse and final crater formation (∼40–120s). Thus, varying $e_{v}$ from 0 to 0.01 when $e_{s}>0$ only has a limited effect on the maximum vibrational velocity during the early stages of modification. On the other hand, the percentage of distortional energy converted to vibrations strongly affects the magnitude of the maximum vibrational velocity, particularly at late times.

**Figure 6:**
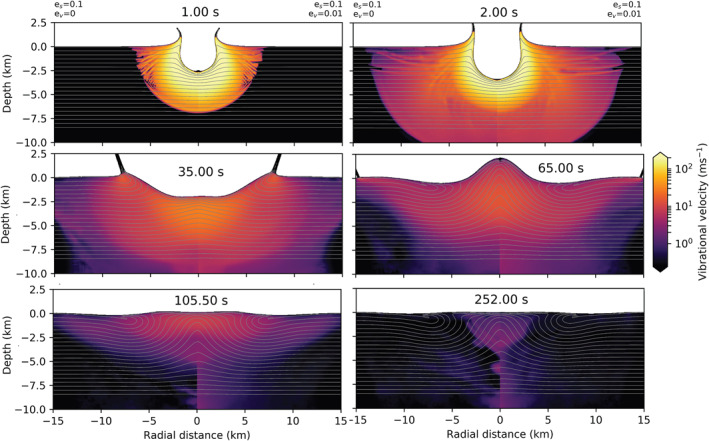
Time series showing the effect of the shear regeneration term $e_{s}$ on crater collapse and evolution of the vibrational field. Left from the origin, $e_{v}$ = 0, and right, $e_{v}$ = 0.01. The origin denotes the point of impact. The impact conditions are the same in both simulations (a = 720 m, $V_{i}$ = 12 km/s). In both models λ = 105 m, *Q* = 431, $e_{s}$ = 0.1, and ξ = $3\times 10^{4}m^{2}$/s.

In Figure 6, we compare the evolution of the vibrational velocity field for simulations with different regeneration parameters. On the left from the origin are results from the simulation that included only the shear regeneration term ($e_{s}=0.1$, $e_{v}=0$), and on the right are results from the simulation that included both shear and volume regeneration ($e_{s}=0.1$, $e_{v}=0.01$). Initially, the generated vibrational field is the same. Slight differences occur in the depth of strong vibrations (≥50 m/s) at 2 s after impact. Between 35 and 65 a tremendous amount of floor collapse occurs in both cases (c.f., Figure 4), leading to a final crater that is too shallow. This suggests that either the shear regeneration parameter is too large or the rate of acoustic dissipation should be increased (e.g., by decreasing Q), or both, to compensate for the stronger vibrational energy field. In our companion paper (Rajšić et al., 2024), we show the trade‐off between the Melosh model parameters $\eta_{\text{eff}}$ and Q, and Q and $e_{s}$. When shear regeneration of vibrations is accounted for, the decay of vibrations, controlled by Q, should be faster because, unlike in the Block model, the vibrations are regenerated during crater collapse. This is the main difference between the two models. In the Block model, the initial vibrational field needs to remain in the target for a longer time, while in the Melosh model, it can decay faster but regenerates throughout the cratering process (Figure 5). After crater collapse, at ≥100 s, stronger vibrations are still present at a greater depth in the case with $e_{v}=0.01$, but the final crater morphology is unaffected.

Our simulations suggest that including shear regeneration of acoustic energy has an important influence on transient crater collapse. The spatio‐temporal evolution of the vibrational velocity field and the duration and degree of crater collapse are very different for simulations with (Figure 4, $e_{s}=0.1$) and without (Figure 6, $e_{s}=0$.) shear regeneration. When shear regeneration is accounted for $\left(e_{s}>0\right)$, however, the addition of the volume regeneration term $\left(e_{v}\right)$ does not affect final crater morphology or temporal evolution of maximum vibrational velocity. This implies that if shear regeneration is a significant source of acoustic energy during crater formation, then vibrations initially generated from compression play a minimal role. In our companion paper (Rajšić et al., 2024), we show that simulations of crater formation using the Melosh model with only shear regeneration ($e_{s}>0$, $e_{v}=0$) can produce a good match to the observed size‐morphology progression of craters on Earth and the Moon. Hence, in the following section, we use the best‐fit Melosh model parameters from that work in simulations of simple, complex, and peak ring craters to highlight the differences in vibrational velocity field evolution between the Melosh and Block models.

### 4.5 Evolution of the Acoustic Vibrations in the Melosh and Block Model

After successfully reproducing similar behavior between the Block and Melosh models that neglected shear regeneration and scattering terms, we expand our modeling to simple, complex, and peak ring craters and directly compare the evolution of acoustic vibrations. In the companion paper (Rajšić et al., 2024) we explore the effects of the decay time of acoustic vibrations, target effective viscosity, shear regeneration, and scattering terms, on a series of numerical models, and define a set of parameters that successfully reproduces simple‐to‐complex transition on the Moon (λ=0.2a, Q=50, $\xi =10^{3}$ m^2^/s, $e_{s}$ = 0.025). We use these parameters in the simulations presented in this section to highlight the differences in the kinematics of crater collapse from the Melosh and Block model. For the Block model, we keep acoustic fluidization parameters constant, with changing $T_{\text{off}}$ according to the size of the target (the smallest scenario, $T_{\text{off}}$ = 0.4 s; in the largest scenario, $T_{\text{off}}$ = 16 s). As described in a previous section, to allow vibrational energy to be generated at late times by shear generation, we set $T_{\text{off}}$ equal to the simulation end time for the Melosh model simulations. We keep impact velocity constant in our models ($V_{i}$ = 12 km/s) and vary impactor radii, a=0.072, 0.72, 7.2 km.

**Figure 7:**
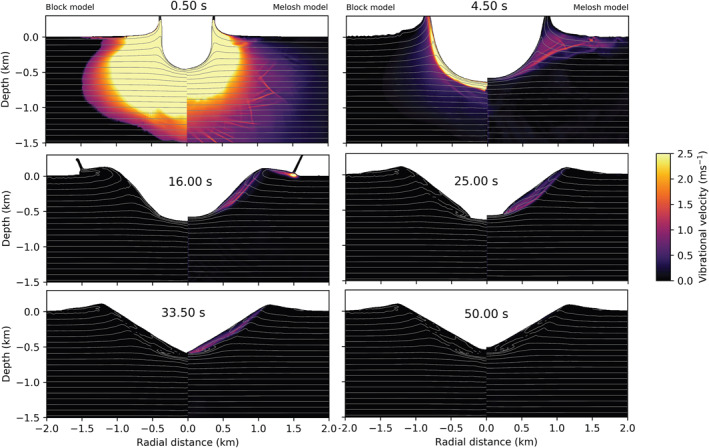
Time series showing the effect of the acoustic fluidization models on crater collapse and evolution of the vibrational field in the smallest impact scenario (a = 0.072 km). Left from the origin simulation that included the Block model ($T_{\text{dec}}$ = 1.66 s; $\eta_{\text{eff}}$ = $7.5\times 10^{6}$ Pa s), and right is a simulation with fiducial parameters for the Melosh model. The origin denotes the point of impact.

In Figure 7, we compare the time series from the Block model simulation (left) to those from the Melosh model simulation (right) for the a=0.072 km impact scenario. In this case, the decay time and effective viscosity of the fluidized region are 10 times smaller than in the mid‐size scenario. Acoustic vibrations in the Block model do not show localization at any time. In both cases, vibrations have the highest magnitude at the first frame as the initially high vibrational velocities imparted by the shock decay after its passage. At t=16–33.5 s, the formation of the breccia lens is shown. In the Melosh model, regenerated vibrations are present in the collapsing material, while in the Block model, all vibrations have ceased. In the last frame, the final crater morphology is shown, where in both cases, all vibrations have ceased, and the depth (in both models ∼0.6 km) and diameter are comparable (Melosh: *D* = 2.4 km; Block: *D* = 2.6 km).

**Figure 8:**
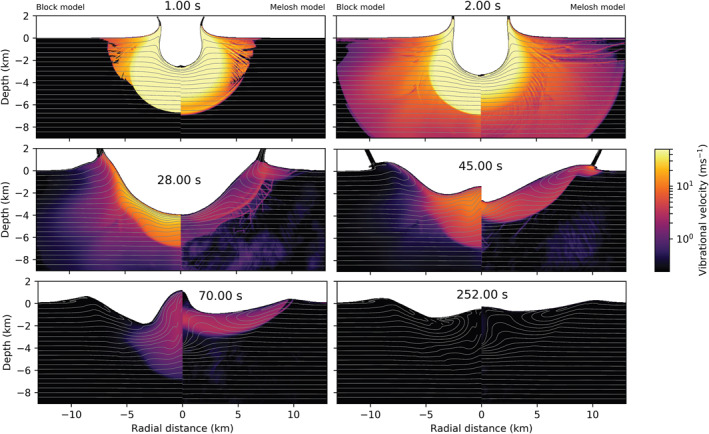
Time series showing the effect of the acoustic fluidization models on crater collapse and evolution of the vibrational field in the mid‐sized impact scenario (a = 0.72 km). Left from the origin simulation that included the Block model, and right is a simulation with fiducial parameters for the Melosh model. The origin denotes the point of impact. Note different scales for the first 2 s after impact and later times.

In Figure 8, we compare the Block (left) and Melosh model (right) simulations for our mid‐size scenario (a = 0.72 km). Initially, the vibrations are the strongest in the first time frame (*t* = 1 s). The differences between vibrational velocity fields in the models are noticeable 2 s upon the impact, whereas in the Melosh model, vibrations already show localization near the surface. Progressively, vibrations slowly decay during crater formation in the Block model and more rapidly in the Melosh model (*t* = 28 s). During crater floor uplift (*t* = 28–70 s), there are three main differences: (a) the vibrations in the Melosh model are regenerated during this stage, while the strong vibrations generated at the early stages of crater formation in the Block model are still decaying (*t* = 28 s) (b) vibrations' origin in the Melosh model is ∼2 times shallower than in the Block model (*t* = 45 s); (c) at *t* = 70 s the vibrations in the Melosh model are wider in the lateral direction, localizing at the crater rim. At the same time, in the Block model, vibrations still exist in the central uplift but have ceased laterally outside of ∼5 km. In the last frame, we show the final crater morphology. Crater diameter in both models is similar: in the Block model ∼18 km, and in the Melosh model ∼20.5 km. The main difference is that the Block model produces a deeper crater with a higher rim. This is reflected in different rim‐to‐floor depths: Block model 1.77 km and Melosh model 1 km.

**Figure 9:**
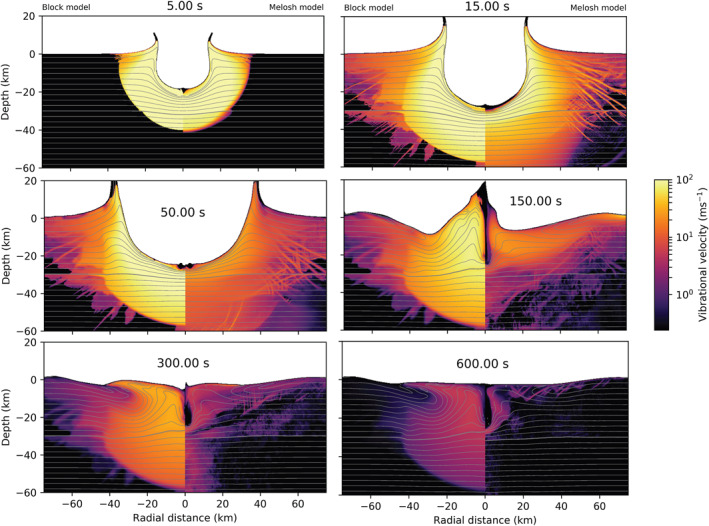
Time series showing the effect of the acoustic fluidization models on crater collapse and evolution of the vibrational field in the largest impact scenario (a = 7.2 km). Left from the origin simulation that included the Block model ($T_{\text{dec}}$ = 165.6 s; $\eta_{\text{eff}}$ = $7.5\times 10^{8}$ Pa s), and right is a simulation with fiducial parameters for the Melosh model. The origin denotes the point of impact.

Finally, we compare two simulations for our largest impact scenario (a = 7.2 km; Figure 9). In this case, the decay time and effective viscosity of the fluidized region are 10 times larger than in the mid‐size scenario. Due to the impact size, the largest impact scenario included a 30 km thick crust and mantle with dunite equation of state (Benz et al., 1989). These simulations also included a thermal gradient of 10 K/km and conductive lid thickness 100 km (e.g., Rae et al., 2019). For simplicity, the same acoustic fluidization model parameters are adopted in both the crust and mantle.

Shortly after impact (top frames), vibrational velocities are strong in both cases. However, in the Melosh model, vibrations are more localized, have a wide lateral extent, and at *t* = 15 s have lower magnitudes at depth. During the crater floor collapse (t=50–150 s), the vibrations in the Melosh model are regenerated and more localized. At the same time, in the Block model, strong vibrations still remain deep in the target (∼ 60 km below the surface). However, their lateral extension is reduced. The radial width of the uplifted material is 3× larger in the Block model simulation than in the Melosh model, but the final crater morphology is similar (*t* = 600 s). Crater in the Block model is 2% larger (*D* = 143.6 km) than in the Melosh model, and their depth‐to‐diameter ratio is comparable, 0.025 (Block model) and 0.027 (Melosh model). The main difference is that the peak ring is more pronounced in the Block model due to a greater volume of material being uplifted.

The main difference between the acoustic energy field in the Melosh and Block models (Figures 7, 8, 9) is that when shear regeneration is included, the Melosh model requires a six‐times‐shorter decay time of acoustic vibrations to produce same crater morphology as the Block model. This helps to resolve a limitation of the Block model. Ivanov and Artemieva (2002) describe how the Block model parameters $\eta_{\text{eff}}$ and $T_{\text{dec}}$ can be related to physical properties of the fluidized rock mass, including block size (h), bulk sound speed $\left(c_{B}\right)$, and quality factor (Q). According to this model, Q can be expressed as:

(9) $$Q=\frac{T_{\text{dec}}\rho c_{B}^{2}}{2\pi \eta_{\text{eff}}}\left(\frac{\rho_{b}}{\rho }\right)\left(\frac{h}{h_{b}}\right),$$

where $h_{b}$ and $\rho_{b}$ are the thickness and density of the inter‐block breccia, respectively. Under the assumptions $\rho_{b}/\rho =0.5$ and $h/h_{b}=10$, this relationship simplifies to:

(10) $$Q\approx \frac{T_{\text{dec}}\rho c_{B}^{2}}{\eta_{\text{eff}}}=\frac{\gamma_{\beta }}{\gamma_{\eta }}{\left(\frac{c_{B}}{c_{p}}\right)}^{2}.$$

Thus, for the Block model simulations discussed here, $Q\approx 14375{\left(c_{B}/c_{p}\right)}^{2}$. To reconcile these Block model parameters with typical seismically derived values (Q∼50–600 for the uppermost crystalline rocks, Bradley & Fort Jr, 1966; Waters & Waters, 1981) would require an unrealistically low bulk sound speed of the fractured and fluidized rock mass that is less than one‐tenth of the target's initial sound speed $c_{p}$. For a more plausible reduction in sound speed of a factor of two, the Q‐value implied by the Block model parameters is 3,600. This is consistent with previous work (Rae et al., 2017) that concluded that the Block model parameters that produced the best match to observations from the West Clearwater Lake impact structure implied a Q‐value more than an order of magnitude larger than typical seismically derived Q‐values. The high implied Q‐value of the Block model compares with a value of Q=50 adopted in our simulations with the Melosh model. Hence, a realistic temporal decay of acoustic vibrations in impact simulations appears to require the dynamic regeneration of acoustic energy.

On the other hand, the Block model simulation produces a more pronounced peak ring (Figure 9), which agrees better with the currently favored mechanism of peak ring formation (Collins et al., 2002; Morgan et al., 2000, 2011). In the companion paper (Rajšić et al., 2024), we explore the trade‐off between Q and $e_{s}$ and show that increasing $e_{s}$ results in greater localization of acoustic energy, which helps enhance peak ring formation. It may also be necessary to consider spatial variation of Q within the crust and upper mantle (as well as differences in Q between planetary bodies). Larger impacts will fluidize deeper material where the characteristic Q is likely higher. This may lead to an effective increase in Q with impact size, extending the fluidization duration during central uplift formation, collapse, and peak‐ring formation.

## 5 Conclusions

Dramatic weakening of rock is required to explain the morphology of large, complex impact craters. Melosh (1979) derived a strength‐weakening model known as acoustic fluidization to explain this phenomenon, where temporary reduction of a material's frictional shear strength occurs due to pressure fluctuations. Here we modified the iSALE shock physics code to include the full nonlinear model of acoustic fluidization. We compare the strength‐weakening characteristics of the Melosh model to a widely used alternative, the block‐oscillation model (Ivanov & Kostuchenko, 1997).

By neglecting scattering and shear regeneration in the Melosh model, we generated an acoustic energy field consistent with the Block model, assuming that 1% of specific internal energy increase from PdV work is converted to specific acoustic energy. This resulted in qualitatively the same process of complex crater collapse in simulations using both the Melosh and the Block model. The final morphology of the craters in each case was very similar, with comparable depth/diameter ratios of d/D = 0.1 (Block model) and 0.09 (Melosh model).

Further, we explored the effects of including terms governing the spatio‐temporal evolution of acoustic energy that are neglected in the Block model. Including the scattering term in the simulations resulted in a broader but weaker zone of fluidization. However, the final morphology of the craters was not affected. Including shear regeneration of acoustic energy, on the other hand, produced a larger and more sustained fluidized region, resulting in more dramatic crater collapse.

Finally, we applied Melosh model parameters that have been found to reproduce the crater size‐morphology progression on Earth and the Moon (Rajšić et al., 2024) to three impact scenarios of different scales: simple, complex, and peak ring craters. In each case, we compared the crater formation and evolution of the vibrational field between the Melosh model and the equivalent best‐fit Block model simulation. In all explored cases, crater collapse in the Melosh model is facilitated by the shallow regeneration of localized acoustic energy. In contrast, collapse in the Block model results from long‐lived vibrations extending deep into the target. Localized, shallow acoustic vibrations in the Melosh model result in different styles of sub‐crater deformation between the two models. In the Melosh model, crater collapse is sustained by substantial regeneration of acoustic energy during crater growth and collapse rather than by slow dissipation of acoustic energy to heat. This helps to reconcile very long vibration decay times and correspondingly large Q‐values implied by the Block model with typical seismically derived Q‐values that are much lower.

Our work, together with the companion paper (Rajšić et al., 2024), shows that the Melosh model of acoustic fluidization leads to qualitatively different kinematics of crater collapse compared with the well‐established Block model. Further simulations and careful comparison with geological, geophysical and remote sensing observations are required to explore which of the two models of acoustic fluidization is best able to reproduce subsurface deformation in complex craters and impact basins.

## Data Availability

All simulations presented in this work were made using the iSALE Dellen version (Collins et al., 2016). iSALE is available for use by researchers in planetary science, although it is strictly for non‐commercial use. Researchers can request iSALE access via the application form (https://isale‐code.github.io/access.html). Input files used in simulations presented in this work are available at the Zenodo repository Rajšić (2024).
